# Epigenetic Regulation of Chromatin Functions by MicroRNAs and Long Noncoding RNAs and Implications in Human Diseases

**DOI:** 10.3390/biomedicines13030725

**Published:** 2025-03-16

**Authors:** Salvatore Costa, Gaspare La Rocca, Vincenzo Cavalieri

**Affiliations:** Department of Biological, Chemical and Pharmaceutical Sciences and Technologies (STeBiCeF), University of Palermo, Viale delle Scienze Bld. 16, 90128 Palermo, Italy

**Keywords:** miRNA, long noncoding RNA, circular RNA, histone modifiers, epigenetics, chromatin, disease

## Abstract

The bulk of RNA produced from the genome of complex organisms consists of a very large number of transcripts lacking protein translational potential and collectively known as noncoding RNAs (ncRNAs). Initially thought to be mere products of spurious transcriptional noise, ncRNAs are now universally recognized as pivotal players in cell regulatory networks across a broad spectrum of biological processes. Owing to their critical regulatory roles, ncRNA dysfunction is closely associated with the etiopathogenesis of various human malignancies, including cancer. As such, ncRNAs represent valuable diagnostic biomarkers as well as potential targets for innovative therapeutic intervention. In this review, we focus on microRNAs (miRNAs) and long noncoding RNAs (lncRNAs), the two most extensively studied classes in the field of ncRNA biology. After outlining key concepts of miRNA and lncRNA biogenesis pathways, we examine their multiple roles in mediating epigenetic regulation of gene expression and chromatin organization. Finally, by providing numerous examples of specific miRNAs and lncRNAs, we discuss how dysregulation of these mechanisms contributes to the onset and/or progression of various human diseases.

## 1. Introduction

The epigenetic machinery comprises a complex network of gene regulatory mechanisms that operate on chromatin without involving changes in genome sequence [1]. Epigenetic regulation operates through DNA methylation, histone post-translational modifications and the replacement of canonical histones with specialized histone variants, nucleosome positioning and density, three-dimensional chromatin organization, and ncRNAs [2,3].

Although RNA was pioneeringly proposed in 1975 to act as a structural component of chromatin [4], transcripts lacking clear protein-coding potential were predominantly regarded as transcriptional noise or mere byproducts of transcription [5]. The critical regulatory roles of ncRNA began to emerge over the past two decades or so, when the deep sequencing of transcriptomes isolated from prokaryotic and eukaryotic organisms revealed that pervasive genome transcription is a universal occurrence [6,7].

The plethora of ncRNAs currently known is arbitrarily categorized into small and long ncRNAs based on their size, with a 200-ribonucleotide threshold, a standard cutoff commonly used in RNA isolation protocols, serving as the dividing line [8]. By specifically interacting with DNA, proteins, and other RNA molecules, ncRNAs directly or indirectly control multiple epigenetic regulatory layers. This occurs in a spatiotemporal manner as ncRNAs move across the nucleoplasm and throughout the cell during their lifetime, impacting various key biological functions [9]. Given the impact of ncRNAs on these cellular processes, it is not surprising that dysregulation of ncRNA functions has been widely implicated in a multitude of disease states, including cancer [10,11,12].

Currently, research in the field of epigenetics and advanced molecular biology is strongly focused on identifying and functionally characterizing the full spectrum of ncRNAs involved in physiological functions, their crosstalk with other epigenetic mechanisms, their role in the onset of human diseases, and their potential as druggable targets for therapeutic intervention.

In this review, we focus on miRNAs and lncRNAs, the two most extensively studied classes in the field of ncRNA biology. We first outline key concepts of their biogenesis pathways and then explore their diverse roles in regulating gene expression and chromatin organization through epigenetic mechanisms. At the same time, we illustrate how the dysregulation of these mechanisms contributes to the onset and progression of various human diseases by presenting numerous examples of specific miRNAs and lncRNAs.

## 2. miRNAs

### 2.1. Canonical Biogenesis and Mechanisms of Action of Cytoplasmic miRNAs

This section focuses on miRNAs, the most widely studied and known class of regulatory ncRNAs. miRNAs are evolutionarily conserved single-stranded molecules with an average length of 22 ribonucleotides in their mature form [13,14]. While the exact number of experimentally validated miRNAs is subject to ongoing refinement, current estimates suggest the presence of several hundred miRNAs derived from the human genome [15]. Within the genome, miRNA loci are located either in intergenic regions or embedded within genes and are frequently organized into clusters, producing primary transcripts that contain the genetic information for multiple mature miRNAs [16,17].

In metazoans, most mature miRNAs are generated through the canonical biogenesis pathway of miRNAs (thoroughly reviewed in [18,19,20]), which involves the sequential cleavage of long transcripts first by the nuclear RNaseIII DROSHA, followed by a second cleavage mediated by the cytoplasmic RNaseIII DICER. The mature miRNA is then loaded into the effector complex known as the miRNA-induced silencing complex (miRISC) [21]. Within the miRISC, a member of the Argonaute (AGO) protein family retains the miRNA, facilitating its interaction with a specific mRNA target through Watson–Crick base-pairing [22]. The regulatory mechanism of miRNAs depends on the degree of sequence complementarity between the miRNA and the mRNA: a perfect base match typically results in direct cleavage and degradation of the target, while incomplete annealing is associated with suppression or, less commonly, stimulation of target translation as well as a reduction in target half-life through decapping and deadenylation [23,24,25,26]. This latter mode of mRNA repression is the one widely employed in metazoans and relies on the recruitment of TNRC6 proteins on the miRISC [27]. Notably, each miRNA can theoretically regulate hundreds of distinct mRNAs that share local sequence similarity, while a single mRNA might be modulated by multiple miRNAs [28,29]. Owing to this remarkable promiscuity, miRNAs are estimated to regulate about one-third of human genes in a spatiotemporally specific manner, thereby impacting the homeostasis of cells, tissues, and organs [30].

### 2.2. Regulatory Mechanisms of Nuclear miRNAs

**Figure 1:**
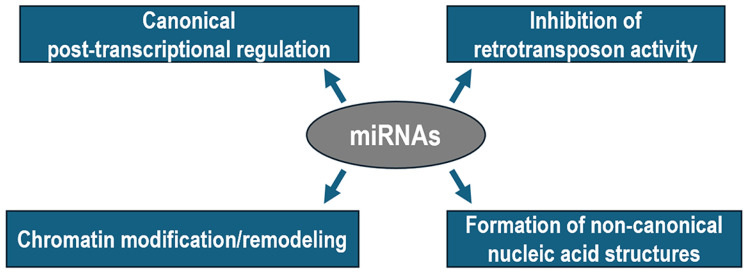
Diagram illustrating the canonical and non-canonical functions of miRNAs in metazoans.

Mounting evidence suggests that miRNAs are differentially represented in distinct subcellular compartments. Indeed, beyond their conventional and well-established roles in the cytoplasm, mature miRNAs can re-enter the nuclear compartment of mammalian cells, where they are implicated in direct epigenetic regulation of target genes [31,32] (Figure 1). Interestingly, some miRNAs are naturally enriched in the nucleoplasm of distinct cell types and specifically adjust their nuclear abundance in response to environmental stimuli [33,34]. For example, the levels of a group of 13 miRNAs were reported to increase, while those of another group of 35 miRNAs decreased, in the nucleus of endothelial cells after hypoxia [35].

Nuclear translocation of miRNAs is a highly regulated and dynamic phenomenon that requires two simultaneous conditions to occur. First, miRNAs must have a hexanucleotide nuclear localization motif at their 3′ terminal sequence, which is thought to be recognized by nuclear pore components directly involved in the transport process [36]. Second, miRNAs must be loaded by an AGO protein, allowing for the resulting complex to shuttle from the cytoplasm to the nucleus and back again by the mediation of IMPORTIN-8 and EXPORTIN-1, respectively [37,38].

Once inside the nucleus, miRNAs either stimulate or suppress transcription of target genes through distinct mechanisms, depending on the genomic location of the target region. More frequently, miRNAs can directly bind to complementary sequences of single-stranded DNA that normally forms after the melting of the basal gene promoter during transcription initiation. For example, miR-195 associated with AGO2 recognizes the TATA box at the *foxO3* gene promoter, facilitating the recruitment of chromatin remodeling complexes responsible for hypomethylation of lysine 9 of histone H3 (H3K9), histone acetylation, and transcriptional activation of *foxO3* in ovarian granulosa cells [39]. Indirect evidence obtained by using synthetic small activating RNAs against the progesterone receptor gene promoter suggests that the genomic target site may map far upstream of the basal promoter [40]. Similar mechanisms are exploited by miR-223 to promote *NFI-A* gene silencing, enabling granulopoiesis in human hematopoietic progenitors [41]. Specifically, a miR-223–DNA hybrid duplex forms twice within a chromatin region spanning two nucleosomes upstream of the *NFI-A* transcription start site [41]. This interaction favors the recruitment of the Polycomb Repressive Complex 2 (PRC2), which trimethylates lysine 27 of histone H3 (H3K27me3) to promote chromatin compaction and gene silencing [41].

According to recent studies, nuclear miRNAs bind not only to promoters but also to enhancers to induce the expression of both neighboring and distantly located genes [42]. Mechanistically, the interaction between the miRNA and the complementary enhancer sequence triggers the recruitment of histone-modifying complexes that reduce the level of the repressive mark H3K27me3 while increasing those of acetylated H3K27 and monomethylated H3K4, both associated with active enhancer function [43]. The resulting permissive chromatin environment enables the transcription of a so-called enhancer-RNA, which promotes target gene(s) expression through chromatin looping between the enhancer and promoter aided by MEDIATOR complex recruitment or COHESIN assistance [44].

Computational predictions suggest that miRNAs may contribute to the formation of multistranded non-canonical nucleic acid structures, including a hybrid miRNA–DNA triplex. In this case, a given pyrimidine-rich miRNA could directly interact with a purine-rich stretch of duplex DNA via Hoogsteen or reverse Hoogsteen hydrogen bonds in the major groove, thereby favoring or disfavoring the accessibility of transcriptional regulators [45]. Furthermore, a very recent report demonstrated that nuclear miR-9 is involved in the formation of hybrid G-quadruplexes at super-enhancers and at the promoters of TGFB1-responsive genes and that these non-canonical structures are essential for chromatin looping, H3K4me3 deposition, and transcriptional activity in lung fibroblasts [46,47,48].

An additional role of the miRNA pathway in the nucleus of mammalian cells has been recently suggested, which is that AGO proteins can recognize and repress retrotransposons in the nucleus of quiescent splenic B cells [49]. Mechanistically, the study showed that, in quiescent cells, when mitogenic signals are low, TNRC6 proteins are minimally expressed, which is sufficient to drive nuclear localization of AGO, thereby favoring the association of the miRNA–AGO complex with the chromatin. Given the potential catastrophic consequences for the genome of unrestrained transposition, this nuclear miRNA function may represent an important mechanism to preserve tissue integrity and animal health.

### 2.3. Roles of miRNAs in Physiological and Pathological Processes

As post-transcriptional regulators of gene expression, miRNAs are typically involved in complex regulatory circuitries that functionally influence key epigenetic players and biological processes (Table 1) [39,41,46,47,48,50,51,52,53,54,55,56,57,58,59,60,61,62,63,64,65,66,67,68,69,70]. Moreover, since alterations in miRNA functions are linked to various human pathologies, targeted modulation of specific miRNA functions has been proposed for therapeutic applications [71]. For example, in myoblasts, miR-214 negatively regulates the expression of the histone methyltransferase EZH2, the enzymatic core component of the PRC2 complex, which establishes an inactive chromatin state by accumulating the repressive H3K27me3 epigenetic mark [50]. This leads to reduced deposition of H3K27me3 at selected chromatin regions, thereby promoting musculoskeletal-specific gene expression and differentiation [51]. Worth mentioning, upregulation of the miR-214 level is strongly correlated with muscle fibrosis in Duchenne muscular dystrophy [52], suggesting that normalization of miR-214 function could represent an attractive therapeutic approach to treat myopathies.

As mentioned, several miRNAs coregulate distinct targets, comprehensively referred to as the targetome, and exhibit remarkable functional pleiotropy, suggesting that their dysregulation can lead to widespread biological consequences. A pertinent example is provided by miR-132, a versatile regulator that plays pivotal roles in diverse physiological processes, including neuronal homeostasis, immune response, tissue repair, lymphopoiesis, and hematopoiesis [72,73,74,75,76]. Down-regulation of miR-132 in the prefrontal cortex is associated with schizophrenia through overexpression of a number of direct targets, including the de novo DNA methyltransferase DNMT3A and the methyl-cytosine-binding protein MeCP2 [53]. Transgenic mouse models revealed that miR-132 is robustly induced by light stimulation in the suprachiasmatic nuclei, where it orchestrates chromatin remodeling of circadian genes by modulating key epigenetic players that, in turn, fine-tune mammalian clock entrainment [54,55]. These epigenetic regulators include MeCP2, the histone acetyltransferase p300, the NAD^+^-dependent deacetylase SIRT1, and the H3K4 demethylase JARID1a [54,55]. Collectively, these findings suggest that deregulation of miR-132 represents a causative factor in the onset of human pathophysiological conditions associated with disturbances in circadian rhythms. miR-132 levels are transiently increased in models of chronic inflammatory conditions and following infection of bacteria and viruses with diverse cell tropism, including human cytomegalovirus, herpes simplex virus-1, and Kaposi’s sarcoma-associated herpesvirus [56,57,58,59]. Under these circumstances, miR-132-induced suppression of *p300* mRNA translation leads to reduced acetylation of the H3 histone, which, in turn, attenuates the transcriptional activation of viral genes, thereby contributing to antiviral response [59]. In striking contrast, miR-132 has been reported to leverage the same epigenetic system in order to potentiate the efficiency of HIV-1 replication rather than restrain it [60]. In any case, the most important implication of these findings is that miR-132 may serve as a powerful therapeutic target for the prevention and control of viral infections.

Numerous miRNAs are markedly dysregulated in carcinogenesis, exerting either tumor-suppressive or oncogenic functions depending on the specific tissue and/or tumor type [77] (Table 1). Several studies have consistently shown that, in most of these pathological conditions, miRNA deregulation contributes to the establishment of abnormal epigenetic landscapes that drive tumor onset, progression, and metastasis [78]. Indeed, in these cases as well, miRNA-deregulated targets code for various histone-modifying enzymes and DNA methyltransferases (Table 1). For example, miR-101 abundance specifically decreases during the progression of multiple types of cancer, paralleling an increase in the expression of the EZH2, DNMT3a, and histone deacetylase (HDAC) 9 enzymes along with global aberrations in the epigenetic marks they regulate [61,62,63].

Understandably, disturbances of regulatory mechanisms governed by nuclear miRNAs are also typically associated with a number of pathological conditions, including cancer. For example, the specific interaction of miR-584 with the *MMP14* gene promoter, normally occurring in gastric epithelial cells, is required to impose gene silencing by favoring the local enrichment of the repressive marks H3K27me3 and H3K9me3 along with a decreased binding of the YY1 transcriptional activator [64]. Downregulation of miR-584 in gastric cancer cells leads to increased *MMP14* expression, the levels of which are directly correlated with tumorigenesis, aggressiveness of gastric cancer, and poor patient survival [64,65]. Similarly, nuclear abundance of miR-339 has been proposed for re-classification of breast cancer subtypes. Indeed, both miR-339 and the tumor suppressor gene *GPER1* are differentially downregulated in distinct breast cancer subtypes [66]. Importantly, transfection of miR-339 specifically re-activates *GPER1* expression by interacting with its enhancer, thereby inhibiting the proliferation of breast cancer cells [66]. It follows that miR-339 could represent a promising cornerstone for the development of an innovative clinical approach to treat breast cancer.

### 2.4. Strategies and Challenges in miRNA-Based Therapeutic Approaches

As mentioned in the previous section, the central role of miRNA function in illness, especially cancer, has made them desirable targets and tools for cutting-edge therapeutic strategies. In particular, miRNA mimics (agomiRs) and RNA molecules that target miRNAs (antagomiRs) have shown promise in preclinical development. Indeed, a number of miRNA-targeted treatments have advanced to the clinical stage, such as miravirsen, the synthetic antagomiR for miR-122, which reached phase II trials for treating HCV-induced hepatitis [79], and MRX34, the agomiR of the tumor suppressor miR-34, employed in phase I clinical trials for the treatment of solid hepatocellular carcinoma [80]. However, finding safe and targeted miRNA therapies is a major challenge due to the unclear role that miRNAs play in influencing cancer biology [81]. In fact, both mentioned trials were closed early due to serious adverse effects that resulted in patient deaths.

The ability of a single miRNA to repress hundreds of distinct mRNAs at once may explain the difficulty in foreseeing the outcomes of miRNA-targeting treatments. Consequently, depending on the type and state of the cell, miRNA manipulation will produce distinct phenotypes. For instance, a single miRNA can suppress both oncogene and tumor suppressor expression in a cell at the same time, making the outcome on tumor development unpredictable. Depending on the balance and functions of its target genes in that particular cancer type, it is conceivable that altering an miRNA’s expression will either promote or inhibit the growth of a tumor. It could be speculated that cancer cells may be particularly reliant on miRNA-mediated gene regulation while dispensable in post-mitotic tissues so that global loss of miRNA function, as opposed to manipulating individual miRNAs, may be an effective strategy to target cancer. Further work will be crucial to verify this hypothesis at the organismal level.

In conclusion, technical obstacles have prevented miRNAs from moving from the bench to the bedside, despite encouraging findings that suggest a role for miRNAs as biomarkers for cancer diagnosis and targets for the development of anti-cancer therapies.

## 3. lncRNAs

### 3.1. Biogenesis Pathways of lncRNAs

lncRNAs represent the largest and most heterogeneous class of ncRNAs, constituting a substantial fraction of the transcriptome in complex eukaryotic organisms. Recent estimates predict that the human genome produces several tens of thousands of lncRNAs, although their abundances are lower than those of mRNAs [82,83,84]. Generally, lncRNAs are produced in a tissue-specific manner and are characterized by a length ranging from 200 to 1 × 10^6^ ribonucleotides [85]. Moreover, compared to miRNAs, the vast majority of lncRNAs exhibit poor evolutionary conservation, which hampers their identification and annotation across species [86].

LncRNAs are divided into distinct groups according to their genomic location and context [87]. More specifically, lncRNAs are classified as intergenic or intronic, depending on whether their transcription unit is located entirely within an intergenic genomic region or an intron of a gene, respectively. Conversely, when lncRNAs partially or completely overlap the coding region(s) of a gene, they are referred to as sense or antisense lncRNAs, depending on whether they are transcribed from the sense or antisense DNA strand of the gene [86]. Finally, bidirectional lncRNAs arise from transcription units located nearby a gene but are transcribed from the opposite strand.

Unlike the majority of miRNAs, which are univocally produced through a well-defined biogenesis pathway, lncRNAs undergo diverse processing trajectories that ultimately influence their half-life, structure, and subcellular localization [88]. First and foremost, RNA polymerase II can synthesize primary lncRNAs from transcription units embedded in chromatin regions decorated by either permissive or repressive histone modifications [89,90]. These transcripts typically endure the same processing machinery as mRNAs, including 7-methylguanosine capping, constitutive or alternative splicing, and the addition of a 3′ poly-A tail [91]. However, these transcripts are inefficiently spliced and polyadenylated due to weak sequence elements recognized by the processing machinery [90], making them susceptible to degradation by nucleases of the post-transcriptional surveillance system [92]. Taken together, these facets may account for the preferential nuclear retention and low abundance of several mature lncRNAs. On the other hand, lncRNAs are not necessarily processed through the mentioned passages, including those transcribed from RNA polymerase I or III promoters and circular RNAs (circRNAs), which completely lack the 5′ cap and 3′ poly-A tail [93,94].

Within the lncRNA family, circRNAs are distinctive members characterized by a single-stranded covalently closed loop structure that results from a unique processing mechanism known as back-splicing [95]. Interestingly, this process occurs on both noncoding transcripts and pre-mRNAs, where a 5′ splice site is joined, in a reverse order as usual, to an upstream 3′ splice site by the spliceosome machinery [95]. It follows that the composition of circRNAs consists of either one or more exons, although their translational potential remains controversial [96,97]. Less frequently, circRNAs are derived from exonucleolytic degradation of intron lariats excised as byproducts of canonical splicing [98]. Generally, the abundance of circRNAs does not correlate with the efficiency of their biogenesis pathway, which is scarce because back-splicing and canonical splicing are catalyzed by the same molecular apparatus and, therefore, directly compete with each other [99]. Nevertheless, due to their closed structure, which lacks free ends, circRNAs can avoid exonucleolytic cleavage, making them significantly more stable than their linear counterparts and allowing for them to accumulate in relatively high quantities within cells [100,101].

After nuclear processing, whatever the mechanism may be, mature lncRNAs form distinct ribonucleoprotein complexes that ensure either their nuclear retention or export to the cytoplasm via nuclear pore complexes [102]. In particular, *cis*-elements within lncRNA sequences are specifically recognized by RNA-binding factors that confine their localization to precise nuclear or cytoplasmic bodies, where their functional roles are executed [103].

### 3.2. Regulatory Mechanisms and Roles of lncRNAs in Physiological and Pathological Processes

**Figure 2:**
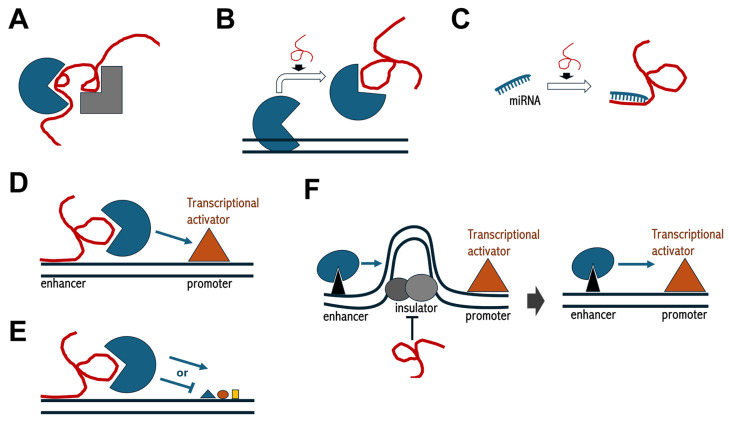
Schematics of regulatory functions mediated by lncRNAs. (**A**) lncRNAs can function as scaffolds to enable protein–protein interactions; (**B**,**C**) lncRNAs can operate as decoys either for proteins or miRNAs, respectively; (**D**) lncRNAs can mediate enhancer functions; (**E**) lncRNAs can recruit chromatin-modifying complexes; (**F**) lncRNAs can interfere with insulator activity, thereby allowing for enhancer–promoter interactions. In each panel, the red convoluted line indicates lncRNA, black parallel lines in (**B**,**D**–**F**) indicate double strand DNA, geometric shapes indicate protein factors, while smaller geometric shapes in (**E**) indicate histone post-translational modifications.

Mechanistically, lncRNAs exhibit different regulatory repertoires (Figure 2) [104]. For example, they can function as scaffolds for protein complexes, bringing together different components into ribonucleoprotein particles to modulate gene expression (Table 2) [105,106,107,108,109,110,111,112,113,114,115,116,117,118,119,120]. In this regard, *lnc-MAP3K13-7:1* serves as a protein-binding scaffold inducing ubiquitin-mediated degradation of DNMT1, which, in turn, leads to hypomethylation of the *cdn1a* gene promoter in granulosa cells [106]. Alteration of this function due to *lnc-MAP3K13-7:1* upregulation causes granulosa cell cycle arrest in the G_0_/G_1_ phase in patients with polycystic ovary syndrome [106]. A similar mechanism is employed by the *jpx* lncRNA, which functions as a scaffold molecule by simultaneously interacting with the phosphorylated p65 subunit of the NF-kB family and the BRD4 histone acetyltransferase to form a remodeling complex involved in nucleosome eviction, chromatin decompaction, and the expression of senescence-associated secretory phenotype genes [107]. Since this epigenetic mechanism is linked to the regulation of cellular senescence, *jpx* could be an attractive therapeutic target for the treatment of age-related atherosclerosis. Another example is circRNA *CircCGNL1*, which interacts with the phosphatase NUDT4 to promote HDAC4 dephosphorylation, leading to its translocation into the nucleus during pancreatic cancer progression [108].

Several lncRNAs can either recruit or change the recruitment of chromatin-modifying proteins to their specific genomic targets, modulating chromatin states and influencing the expression of nearby genes [121]. *HOTAIR*, *PVT1*, *NEAT2*, and several other lncRNAs that recruit Polycomb repressive complexes are typical examples of this mechanism of action [109,110,111]. Although the extent to which lncRNAs contribute to PRC2 chromatin targeting remains debated due to the low specificity of PRC2-lncRNA interaction [122], it is accepted that PRC2 strictly relies on lncRNAs for efficient chromatin binding [123]. For example, the repression of *HOTAIR* target genes coincides with PRC2 promoter occupancy and H3K27 trimethylation across various cell types, regulating several biological processes, including adipocyte differentiation and skin regionalization over the surface of the body [110]. Moreover, silencing of the *dlx1* gene in thyroid cancer cells occurs due to *HOTAIR*-dependent recruitment of PRC2 to the promoter and consequent H3K27me3 deposition, which ultimately increases proliferation, colony formation, and migration of cells [109]. Thus, *HOTAIR* abundance could serve as a novel biomarker for assessing progression and malignancy in thyroid carcinomas.

The lncRNA *NEAT2*, also known as *MALAT1*, plays an elegant role in regulating cell cycle gene expression by physically relocating their chromatin from Polycomb bodies, where they are repressed, to speckles, where they are transcribed [112]. Worth mentioning, relocation between these two nuclear corpuscles depends on the methylation status of Pc2, a component of PRC1, as well as the differential interaction of Pc2 with two distinct lncRNAs, *TUG1* and *NEAT2*. While *TUG1* localizes to Polycomb bodies and interacts with the methylated form of Pc2, *NEAT2* resides in speckles and interacts exclusively with the unmethylated Pc2 protein [112]. As expected, alteration of *NEAT2* function is not only specifically involved in a variety of human diseases but is also often linked to disease severity [124]. Indeed, *NEAT2* upregulation accelerates Parkinson’s disease progression by recruiting DNMT1, DNMT3A, and DNMT3B, leading to hypermethylation and transcriptional silencing of the *socs3* gene [113]. Importantly, *NEAT2* silencing improved neurological function and reduced neuroinflammation in neurotoxin-induced mouse models of Parkinson’s disease [113].

Instead of recruiting chromatin modifiers, lncRNAs can act as decoys, sequestering them away from their genomic targets [125]. For example, *lncPRESS1* titrates the histone deacetylase SIRT6 away from the promoter of numerous pluripotency-related genes, thereby keeping the transcriptionally permissive H3K56ac and H3K9ac marks on their chromatin to support the pluripotency of human embryonic stem cells [114]. Conversely, p53-mediated depletion of *lncPRESS1*, which occurs physiologically during differentiation, reverses this mechanism, switching to transcriptional silencing of pluripotency genes and concomitant activation of differentiation genes [114]. Interestingly, depletion of *lncPRESS1* has been associated with different types of lung carcinomas [115]. Under these circumstances, upregulation of the *EMSLR* lncRNA, which is produced by a transcription unit neighboring that of *lncPRESS1*, favors DNMT1-dependent hypermethylation of the *lncPRESS1* promoter [115].

To some extent, the decoy function is the most common regulatory mechanism also employed by several circRNAs. In fact, they can titrate specific proteins or miRNAs, thereby either masking the function of the protein or favoring the expression of the miRNA target, respectively [126]. For example, the *CNEACR* circRNA binds to HDAC7 in the cytoplasm of cardiomyocytes, preventing its entry into the nucleus [116], while the *circRNA_0058097* upregulates HDAC4 by sequestering miR-365a in human endplate chondrocytes [117].

As mentioned in Section 2.2, a subset of lncRNAs, known as enhancer-RNAs, can act as epigenetic transcription enhancers by favoring chromatin looping and enhancer–promoter contact to activate transcription of their target genes [48,127].

Finally, some lncRNAs may also play a crucial role in coordinating chromatin architecture with the function of chromatin insulators [128,129,130]. A relevant example is provided by *Fub-1^HS2^*, which disrupts the blocking activity of the *Fub-1* insulator by transiently remodeling the topological configuration of *BX-C* chromatin [118]. This enables the enhancer-directed spatiotemporal expression of *BX-C* homeotic genes, ensuring proper patterning of the Drosophila embryo [118]. A similar mechanism is employed in human embryonic stem cells by the *STX18-AS1* lncRNA, which locally antagonizes a CTCF-mediated insulating function to elicit adequate *msx1* gene expression [119]. Predictably, impairing this regulatory mechanism leads to pathological conditions, as altered *msx1* gene expression has pleiotropic effects in several tissues and is known to be linked to multiple diseases [131,132,133].

Several approaches have been considered to modulate lncRNA functions pharmacologically, including RNA interference, antisense morpholino oligonucleotides, CRISPR/Cas9-based technology, and small molecules [134]. However, several limitations have hindered the translation of effective treatments to the clinic. First, the precise impact of lncRNAs in disease pathogenesis is largely unknown, despite the fact that numerous studies have been conducted in clinical patients using different disease models. Such a partial understanding of the role of lncRNAs in cellular pathways has prevented the identification of reliable therapeutic targets. Moreover, the technical limitations that apply to any other class of drugs, such as suboptimal delivery and toxicity, have also hindered the development of therapeutical approaches aimed to target lncRNAs. A full understanding of the impact of lncRNAs in basic, translational, clinical, and pharmaceutical sciences—with a focus on their role in genetic, metabolic, infectious, cancer, and age-related diseases—will then require future mechanistic investigations.

## 4. Conclusions

Despite initially being considered non-essential products of the genome, a growing body of research in the last few years has demonstrated that ncRNAs are versatile multi-level regulators in a wealth of essential biological processes. In particular, since the discovery that ncRNAs, such as miRNAs and lncRNAs, regulate gene expression through epigenetic mechanisms and that misregulation of these mechanisms can influence disease development, the ncRNA field has become the object of intensive research. Although tremendous advancements have recently been achieved in this field, further in-depth studies are needed to fully understand the intricacies of the epigenetic circuitries involving ncRNAs and how their effects are inherited across generations. From a bench-to-clinic perspective, insights from these studies will be fundamental in applying our knowledge of ncRNAs to the diagnosis and therapeutic treatment of various human diseases.

## Figures and Tables

**Table 1:** Selected examples of miRNAs with a reported function either in the cytoplasm or in the nucleus, their validated targets, biological contexts, and corresponding references.

miRNA	Target	Biological Context	References
miR-195	*foxO3* gene promoter	Ovarian granulosa cells	[39]
miR-223	*NFI-A* gene promoter	Granulopoiesis	[41]
miR-9	Super-enhancers and promoters of TGFB1-responsive genes	Lung fibroblasts	[46,47,48]
miR-214	*ezh2* mRNA	Myoblasts	[50,51,52]
miR-132	*dnmt3A* and *mecp2* mRNAs *mecp2*, *p300*, *sirt1*, and *jarid1a* mRNAs *p300* mRNA	Prefrontal cortex suprachiasmatic nuclei bacterial- and virus-infected cells	[53] [54,55] [56,57,58,59,60]
miR-101	*ezh2*, *dnmt3a*, and *hdac9* mRNAs	Multiple types of cancer	[61,62,63]
miR-584	*MMP14* gene promoter	Gastric epithelial normal and cancer cells	[64,65]
miR-339	*GPER1* enhancer	Breast cancer cells	[66]
miR-449	*hdac1* mRNA	Colorectal cancer cells	[67]
miR-574	*hdac9* mRNA	Adipocytes	[68]
miR-137	*lsd1* mRNA	Non-small cell lung cancer	[69]
miR-146	*uhrf1* mRNA	Gastric cancer metastasis	[70]

**Table 2:** Selected examples of lncRNAs with their reported function, validated targets, biological contexts, and corresponding references.

lncRNA	Target	Biological Context	References
*lnc-MAP3K13-7:1*	DNMT1	Granulosa cells	[106]
*jpx*	p65, BRD4	Vascular smooth muscle cells	[107]
*circCGNL1*	NUDT4	Pancreatic cancer cells	[108]
*HOTAIR*	PRC2	Adipocytes, skin epithelial cells, and thyroid cancer cells	[109,110]
*PVT1*	EZH2	Primary multiple myeloma cells	[111]
*NEAT2*	DNMT1, DNMT3A, and DNMT3B	Neurotoxin-induced mouse models of Parkinson’s disease	[112,113]
*lncPRESS1*	SIRT6	Embryonic stem cells	[114]
*EMSLR*	DNMT1	Lung cancer cells	[115]
*CNEACR*	HDAC7	Cardiomyocytes	[116]
*circRNA_0058097*	miR-365a	Endplate chondrocytes	[117]
*Fub-1^HS2^*	*BX-C* chromatin	Drosophila embryo	[118]
*STX18-AS1*	*Msx1* gene	Embryonic stem cells	[119]
*Ppp1r1b*	*Myod1* and *Tbx5* gene promoters, EZH2	Myoblasts	[120]

## Data Availability

No new data were created or analyzed in this study. Data sharing is not applicable to this article.
